# Dual Roles of Carbon Quantum Dots from Green Carbon Sources: A Fluorescence Sensor for Fe^3+^ Ions, UV and High-Energy Blue Light Screening

**DOI:** 10.3390/nano15060436

**Published:** 2025-03-12

**Authors:** Lina Zhong, Chang Sun, Xiaomin Zhao, Qinghua Zhao

**Affiliations:** College of Materials Science and Engineering, Huaqiao University, Xiamen 361021, China; lnzhong@hqu.edu.cn (L.Z.); 22013081053@stu.hqu.edu.cn (C.S.); 15356@hqu.edu.cn (X.Z.)

**Keywords:** green carbon sources, Peperomia tetraphylla, carbon quantum dots, sensor for Fe^3+^, UV and HEBL light blocking

## Abstract

It is of great significance to develop carbon quantum dots (CQDs) using green carbon sources, which are cheap, non-toxic and harmless, and further expand their application scopes, e.g., fluorescence sensors, blue light screening. In this study, we have prepared Peperomia tetraphylla-based carbon quantum dots (PT-CQDs) with strong water solubility, good salt resistance, specific quenching reactions and excellent optical properties via a simple one-step hydrothermal method. In one application, PT-CQDs are utilized as a fluorescence sensor due to their high selectivity and sensitivity to ferric ions (Fe^3+^). The limit of detection (LOD) was 2.7 μmol·L^−1^. On the other hand, PT-CQDs/polyvinyl alcohol (PVA) films with excellent ultraviolet- (UV) and high-energy blue light (HEBL)-blocking properties were obtained. The obtained films exhibited a high blue light weight blocking rate of 100% in UV and 80% in HEBL. The concentrations of the composites could also be controlled to achieve the desired light-blocking rate. In addition, the composites were able to absorb blue light and convert it to other forms of light. These properties suggest their potential applications in the development of advanced blue light screening and fluorescence sensors.

## 1. Introduction

The development and application of nanomaterials has penetrated into many fields, such as physics, chemistry, biology and medicine, attracting the attention of many scholars [1,2,3]. Traditional semiconductor quantum dots (SCQDs) have been applied in the fields of imaging and optics [4,5]. However, carbon quantum dots (CQDs) have attracted more attention in the context of optoelectronics, light-emitting diodes, chemical and biosensing, bioimaging, drug delivery, and photo- and electrocatalysis, because of their low toxicity, excellent biocompatibility, stable emission, and potential to replace toxic heavy metals-bearing SCQDs [6]. Additionally, CQDs possess tunable optical properties, and are easily synthesized, producing completely metal-free, safe, green products due to their composition of only C, N, and O. The raw materials used for the synthesis of CQDs can be divided into organic substances and biomass. Organic substances generally contain carboxyl, amino and other groups [7,8,9,10]. Biomass carbon sources are very abundant, such as fruits [11,12], plants [13,14], seeds [15], peels and other biological wastes [16,17]. Compared with traditional organic substances, biomass carbon sources show more advantages, such as sustainability, low cost, abundant sources and high biocompatibility [18,19].

CQDs in solution have been widely used in biological imaging and chemical sensing [6,20,21]. As a sensor, CQDs show the advantages of fast response, simple operation, low cost, high sensitivity and naked eye observation, and have received a lot of attention and research in the context of heavy metal ion detection [18,22]. Iron, as one of the heavy metals, is also an indispensable trace element in an organism. Once its imbalance occurs, it may damage the cellular system and cause diseases such as Alzheimer’s disease, heart failure, metabolic disorders and Parkinson’s disease [18]. Recently, Selvaraju et al. [23] reported that nitrogen CQDs derived from fresh citrus juice could be utilized as fluorescent probes for the highly selective and sensitive detection of Fe^3+^ ions in industrial wastewater. Some CQDs were also able to detect the Fe^3+^ ions by dual fluorescence and ultraviolet (UV) signals. For instance, Ali et al. [24] achieved N-CQDs emitting bright blue fluorescence by using citric acid and ethylenediamine, which were applied to detect Fe^3+^ in aqueous solution, with LOD of 0.07 μmol·L^−1^ and limit of quantitation (LOQ) of 0.22 μmol·L^−1^, respectively. Peperomia tetraphylla is derived from the leaves of Peperomia tetraphylla; its constituents include various alkaloids, flavonoids and volatile oils, and it could also be utilized in CQDs. However, as far as we know, related studies on its use in the detection of Fe^3+^ ions have not been reported. Thus, it is of great significance to synthesize and expand the application of PT-CQDs.

Although CQDs in solution have been widely used in many fields, CQDs in solution need to be stored at low temperature, and there will be aggregation and precipitation during long-term storage, resulting in a decrease in fluorescence intensity. So, many researchers have made solid-state CQDs and developed applications. There is increasing interest in preventing the overexposure of human skin and eyes to ultraviolet (UV) light and high-energy blue light (HEBL), because of its adverse effects on human health, especially in the skin and eyes [25,26]. Most of the UV-C (100–280 nm) and some of UV-B (280–315 nm) in sunlight are absorbed by the Earth’s ozone layer, while the remaining UV-A (315–400 nm) reaches the Earth’s surface. Exposure to high-energy UV radiation is associated with a risk of skin cancers and eye damage. HEBL (400–450 nm) in the visible light range can also have detrimental effects on human health, damaging our eyes and even our mental health. Furthermore, with the increasing use of smart devices and the Internet of Things (IoT), next-generation optoelectronic products such as smart windows and AR displays have come to the fore. However, as reliance on displays and lighting devices increases, so does exposure to blue light radiation. These devices emit HEBL and small amounts of UV light. In short, it is essential to develop UV light-absorbing or -blocking materials to protect our health from these hazardous light sources. At present, few CQDs using natural renewable biomass as raw materials have been applied to UV absorption. Only Xu et al. prepared N-doped CQDs from waste acorn cups and explored using a UV absorber in a polyvinyl alcohol (PVA) film [27]. Xie et al. synthesized silane-doped GQDs (SiGQDs), which have complete UV-blocking [28]. Liu et al. reported on R-CDs prepared from natural renewable rutin, which not only absorbs all UV light, but also does not affect the transmission of visible light [29]. However, few CQDs can block full UV bands and HEBL in visible light simultaneously.

In this study, Peperomia tetraphylla-based carbon quantum dots (PT-CQDs) were prepared from the leaves of Peperomia tetraphylla. Two major applications were demonstrated, one of which was as a fluorescence sensor for Fe^3+^ ions. Further research showed that the PT-CQDs solution represented good selectivity for Fe^3+^ ions with an LOD of 2.7 μmol·L^−1^, reflecting its potential as a sensor for Fe^3+^ ions. Another was used to block UV and HEBL light. Anti-blue light films were established by embedding PT-CQDs in a polyvinyl alcohol (PVA) matrix film, in which PVA was used to disperse PT-CQDs. These films achieved high blue light weight-blocking rates of 100% in UV-A and UV-B and 80% in HEBL, while maintaining adequate transmission in the visible range of 500–750 nm.

## 2. Materials and Methods

### 2.1. Materials

The fresh leaves of Peperomia tetraphylla were purchased from a flower shop. Iron chloride (FeCl_3_·6H_2_O), nickel chloride (NiCl_2_·6H_2_O), cobalt nitrate (Co(NO_3_)_2_·6H_2_O), and cadmium nitrate (Cd(NO_3_)_2_·4H_2_O), and PVA 1799 (the degree of alcoholics is 98–99%, mol/mol) were purchased from Shanghai Aladdin Biochemical Technology Co., Ltd. (Shanghai, China). Lead nitrate (Pb(NO_3_)_2_) was purchased from Xilong Chemical Co., Ltd. (Shantou, China). Zinc nitrate (Zn(NO_3_)_2_·6H_2_O), copper nitrate (Cu(NO_3_)_2_·3H_2_O), magnesium chloride (MgCl_2_·6H_2_O), ferrous sulfate (FeSO_4_·7H_2_O), silver nitrate (AgNO_3_), barium chloride (BaCl_2_·2H_2_O), calcium chloride (CaCl_2_), manganese chloride (MnCl_2_·4H_2_O), cesium nitrate (Ce(NO_3_)_2_·6H_2_O), hydrochloric acid (HCl), sodium hydroxide (NaOH) and sodium chloride (NaCl) were purchased from Sinopharm Chemical Reagent Co. (Shanghai, China). All reagents were of analytical grade without any further purification. Distilled water for experiments was obtained from a Milli-Q HX7080 system. 

### 2.2. Synthesis of PT-CQDs

PT-CQDs were prepared by a simple one-step hydrothermal method [14,30]. The fresh leaves of Peperomia tetraphylla were washed with distilled water and placed in an oven at 100 °C until completely dried, then ground to powder. Then, 0.5 g of powder was weighed into a Teflon-lined stainless-steel autoclave reactor, 30 mL of distilled water was added, and the reaction was carried out at 200 °C for 6 h. After the reactor was cooled to room temperature, an orange PT-CQDs solution was obtained by filtering through normal-temperature filtration via a 0.22 μm microporous membrane. About 25 mL of PT-CQDs stock solution was obtained. The stock solution was placed in a refrigerator and stored at 4 °C. In the spectra experiment, the working solution was obtained by diluting the stock solution 20 times. In order to obtain the solid powder, the stock solution was dialyzed using a dialysis bag with a molecular weight cut-off of 1000 Da for 24 h, and then vacuum-dried in a freeze-dryer.

### 2.3. Preparation of PT-CQDs/PVA Films

PVA (5 g) was dissolved in 45 mL of distilled water at 100 °C. When the solution was cooled to room temperature, a final colorless and transparent solution (10 wt. %) was obtained. Then, the different contents (0.1, 0.3, 0.5, 0.7 wt. %) of PT-CQDs were mixed with the PVA solution under stirring for 0.5 h. Subsequently, the homogeneous solutions were transferred to a round Petri dish (d = 60 mm) or a square Petri dish (240 × 240 mm) and dried at 35 °C for 2 days [26].

### 2.4. Characterization of PT-CQDs

Transmission electron microscopy (TEM) was performed on a Hitachi H-7650 to characterize the morphology of PT-CQDs. X-ray diffraction (XRD) was performed using a Rigaku MiniFlex 600 (Rigaku, Tokyo, Japan). The functional groups and chemical compositions of the PT-CQDs were evaluated by Thermo Scientific K-Alpha X-ray photoelectron spectroscopy (XPS) and a Thermo Nikolet IS50 Fourier (Thermo Fisher Scientific, Madison, WI, USA) transform infrared spectroscope (FT-IR). UV–visible absorption spectra were recorded on a Shimadzu UV-2600i spectrophotometer (Shimadzu, Suzhou, China). Fluorescence spectra and lifetimes were recorded on an Edinburgh FLS920 fluorescence spectrophotometer (Edinburgh, Kirkton Campus, Livingston, UK). The pH was measured by a Mettler–Toledo FiveEasy Plus FE28 pH meter (Mettler Toledo, Shanghai, China). The carbon quantum dots were dried using an FD-1B-50 freeze-dryer from Beijing Boyikang Experimental Instrument Co., Ltd. (Beijing, China). For practical applications involving UV and HEBL blocking from sunlight, mobile phones, and WLEDs, measurement parameters were recorded using an OHSP350 spectrometer (HOPOO Light&Color, Hangzhou, China). Zeta potentials were analyzed on a Malvern Nano-ZS instrument (Malvern Instruments Ltd. (Malvern, UK)).

### 2.5. Luminescence Properties Test of PT-CQDs

The luminescence properties of PT-CQDs were recorded by UV–visible absorption and fluorescence spectra using a 3 mL working solution. All fluorescence spectra were measured at the optimum excitation wavelength of 350 nm in the emission wavelength range of 360–690 nm, with the excitation and emission slit widths of 1.4 nm. All UV–visible absorption spectra were measured in the 200–800 nm range at a medium speed.

To investigate the stability of PT-CQDs at different pH levels, 20 mL of PT-CQDs working solution was transferred into a glass bottle, then different volumes of HCl (2 mol·L^−1^ and 6 mol·L^−1^) and NaOH (5% and 10%, *v*/*v*) solutions were added to adjust the pH of the PT-CQDs solution. Then, 3 mL samples of the above PT-CQDs solutions with different pH values (2.53, 4.82, 6.49, 7.55, 8.44, 10.49, 11.55) were taken to record the fluorescence spectra. To test the salt resistance of PT-CQDs, 2 mL of PT-CQDs working solution was transferred to a colorimetric dish, then different volumes of 6 mol·L^−1^ NaCl were added to adjust the concentration of NaCl in the range of 0~2.5 mol·L^−1^.

### 2.6. Selective and Sensitive Fluorescence Detection of Metal Ions

The sensing studies of PT-CQDs for metal ions were performed by use of fluorescence spectra. The setup conditions of the fluorescence spectrophotometer were consistent with those of the stability tests of the PT-CQDs. To investigate the selective detection of PT-CQDs for metal ions, 1 mL samples of 10 mmol·L^−1^ metal ion solutions (Mn^2+^, Cd^2+^, Co^2+^, Ce^2+^, Zn^2+^, Cu^2+^, Mg^2+^, Fe^2+^, Fe^3+^, Ag^+^, Ni^2+^, Pb^2+^, Ba^2+^ and Ca^2+^) were added to 2 mL of PT-CQDs working solution at room temperature and mixed thoroughly. The results were recorded from the fluorescence spectra. The influence of interfering ions on the sensing ability of the PT-CQDs-based fluorescent probe for Fe^3+^ ions was investigated using a 2 mL PT-CQDs working solution with 1 mL 10 mmol·L^−1^ Fe^3+^ ions in the presence of equal amounts of different interfering metal ion species (Mn^2+^, Cd^2+^, Co^2+^, Ce^2+^, Zn^2+^, Cu^2+^, Mg^2+^, Fe^2+^, Ag^+^, Ni^2+^, Pb^2+^, Ba^2+^ and Ca^2+^).

To calculate the LOD for Fe^3+^ ions, different volumes of 2 mmol·L^−1^ Fe^3+^ were added to 3 mL of PT-CQDs working solution to achieve a concentration of Fe^3+^ ions in the range of 0~100 μmol·L^−1^. Distilled water was used as the blank. The LOD was calculated based on LOD = 3*σ*/*k*, where *σ* was the standard deviation of the blank signal, and *k* was the slope of the liner calibration curve [24,31].

### 2.7. Sensing Mechanism of PT-CQDs for Fe^3+^

To explore the mechanisms of using PT-CQDs as a sensor for Fe^3+^ ions, we used UV–visible absorption spectra, fluorescence spectra, fluorescence decay tests, zeta potential tests and FT-IR spectra. Then, 3 mL of PT-CQDs and 15 μL of 0.01 mol·L^−1^ Fe^3+^ were used to test the UV–visible absorption spectra. The fluorescence lifetimes were measured at the excitation wavelength of 370 nm. Zeta potentials of PT-CQDs were tested at different pH levels with and without 60 μmol·L^−1^ Fe^3+^. The FT-IR spectrum of PT-CQDs with Fe^3+^ ions was measured in the state of 2 mL PT-CQDs working solution mixed with 1 mL 10 mmol·L^−1^ Fe^3+^ after drying with an infrared lamp.

## 3. Results and Discussion

### 3.1. Morphologies and Structures of PT-CQDs

**Figure 1:**
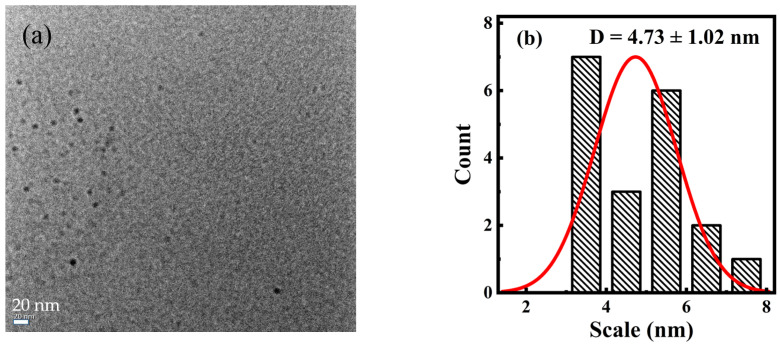

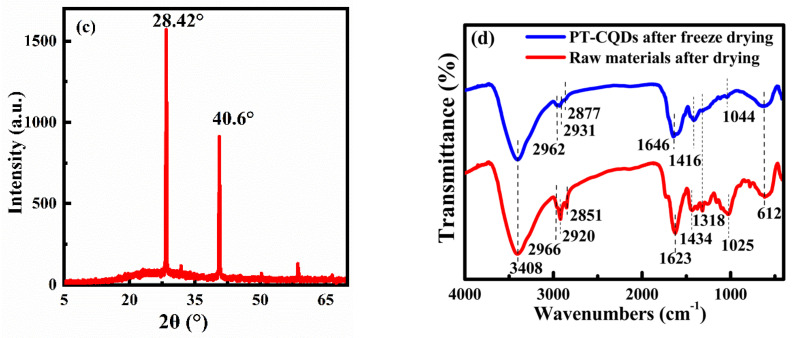
(**a**) TEM image; (**b**) particle size distribution histogram; (**c**) XRD spectrum and (**d**) FT-IR spectra of PT-CQDs after freeze-drying and the leaves of Peperomia tetraphylla after drying (using KBr pressed-disk technique).

TEM was used to perform morphological analyses and study the particle size distribution. The results (Figure 1a) indicate that the synthesized carbon dot PT-CQDs were spherical and monodispersed uniformly in an aqueous solution, which implies that the PT-CQDs had good water solubility. The particle sizes of PT-CQDs were mainly distributed in the range of 4.5~7.6 nm, with an average diameter of about 4.73 ± 1.02 nm (Figure 1b). The XRD spectrum (Figure 1c) showed the crystalline nature of the PT-CQDs, with a broad peak around 25° because of the amorphous carbonaceous form [13]. The two strong and sharp peaks at around 28.42° and 40.6° correspond to (002) and (100) crystal planes of graphitic carbon [32]. The chemical structures of PT-CQDs after freeze-drying and the leaves of Peperomia tetraphylla after drying were determined from the FT-IR spectrum (Figure 1d). For the PT-CQDs, the strong broad absorption band at around 3408 cm^−1^ was attributed to the stretching vibrations of N–H and O–H, indicating that the functional hydroxyl and amino groups were present in the PT-CQDs, contributing to their good water solubility and potential interaction with hard metal ions such as Fe^3+^ iron [11,26,33]. Peaks at 2962 cm^−1^ stemmed from C–H vibrations [16], and 612 cm^−1^ represented the C–H bending vibrations [34]. The strong absorption peak at 1646 cm^−1^ corresponded to the combined stretching vibrations of C=C, C=N and C=O bonds [26,33,34]. The peaks at 1416 cm^−1^ and 1044 cm^−1^ represented the O-C=O and C-N vibrations [35]. Furthermore, from the FT-IR spectrum of the leaves of Peperomia tetraphylla after drying, we can also see several obvious characteristic peaks, including those for the O–H, N–H, C=O, C=C, C=N, O-C=O and C-N bonds. This phenomenon demonstrates that the raw materials after drying and the synthesized PT-CQDs were rich in -COOH, -NH_2_, and -OH.

**Figure 2:**
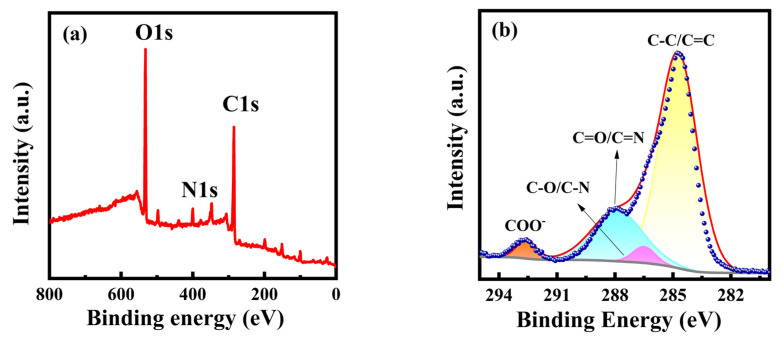

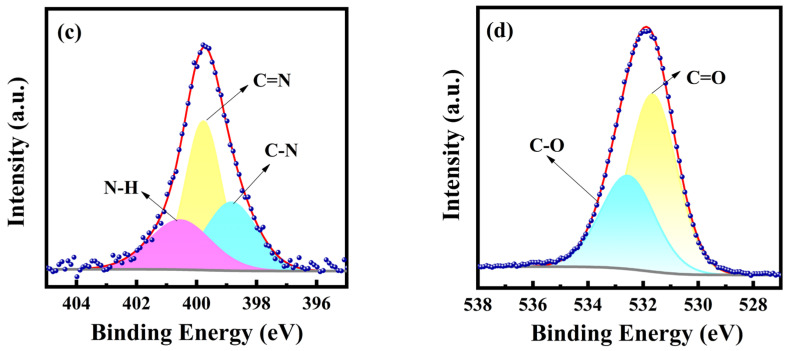
(**a**) XPS survey spectrum and high-resolution XPS spectra of (**b**) C1s, (**c**) N1s, and (**d**) O1s of PT-CQDs.

XPS spectra were further used to analyze the elemental composition and the chemical bonding of PT-CQDs. The full XPS spectrum (Figure 2a) of PT-CQDs shows the characteristic peaks of C1s, N1s, and O1s located at 285.41 eV, 400.19 eV and 532.32 eV, respectively. The relative elemental contents of PT-CQDs were found to be 61.99% (C), 5.40% (N), and 32.61% (O), respectively. The higher contents of C and O confirm that PT-CQDs were rich in oxygen-containing functional groups. The high-resolution XPS spectra of C1s, O1s, and N1s are shown in Figure 2b–d. The high-resolution XPS spectrum of C1s gives four peaks at 284.71 eV, 286.49 eV, 287.88 eV, and 292.58 eV, representing the presence of different types of carbon, C-C/C=C, C-O/C-N, C=O/C=N [35], and COO^−^ [34]. The N1s spectrum exhibits three peaks at 398.84 eV, 399.77 eV and 400.55 eV, derived from C–N, C=N and N–H [36]. The spectra of O1s show two relative oxygen species of C=O (531.65 eV) and C-O (532.56 eV) [11,33]. The XPS spectra confirm that PT-CQDs contained oxygenated and nitrogated groups. The XPS and FT-IR results confirm the presence of oxygenated and nitrogenous groups, such as amino, carboxyl and hydroxyl groups, in PT-CQDs, which enhanced their water solubility and biocompatibility.

### 3.2. Luminescence Properties of PT-CQDs

**Figure 3:**
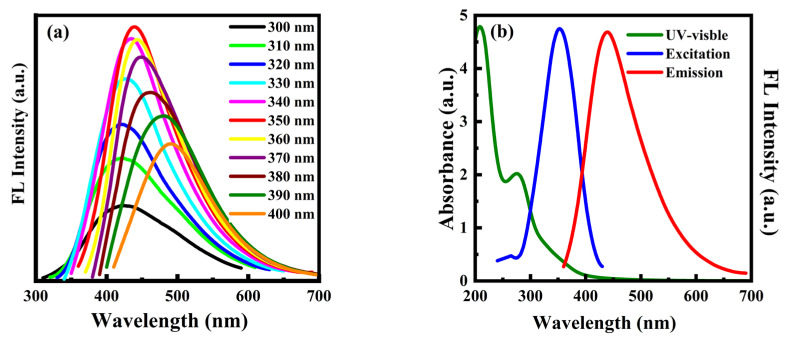
(**a**) Fluorescence spectra of PT-CQDs at various excitation wavelengths. (**b**) UV-Vis absorption spectrum and fluorescence excitation and emission spectra of PT-CQDs.

The optical properties of PT-CQDs were studied by use of UV-Vis absorption spectra and fluorescence spectra. In Figure 3a, the fluorescence spectra of PT-CQDs exhibit red shifts with increasing excitation wavelengths in the range of 300–400 nm. The highest fluorescence intensity of PT-CQDs occurred at the excitation wavelength of 350 nm. As shown in Figure 3b, the UV-Vis absorption spectrum exhibited strong absorption at about 209 nm and a shoulder peak at 275 nm, which were attributable to π–π* and n–π* transitions, respectively [13]; meanwhile, the optimum excitation and emission spectra were at 350 nm and 440 nm, respectively.

**Figure 4:**
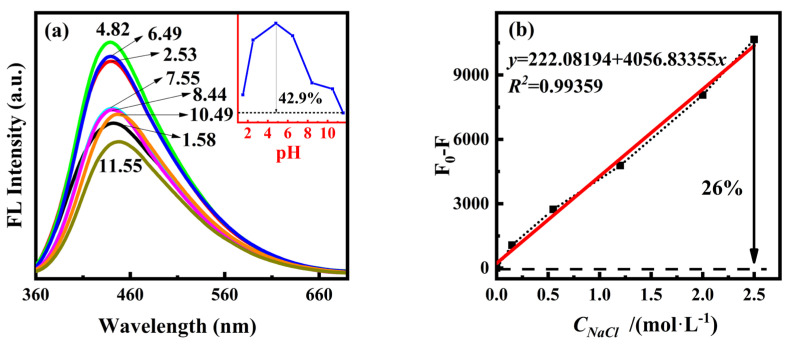
(**a**) Fluorescence spectra and the intensity variation of PT-CQDs in the range of pH 1~12. (**b**) Fluorescence intensity variation of PT-CQDs at different concentrations of NaCl.

To further study the optical properties of PT-CDs, we investigated the effects of pH and high salt concentration on the fluorescence performance of PT-CQDs. The pH of the original working solution of PT-CQDs was approximately 4.82. As shown in Figure 4a, the shape of the fluorescence spectra remained unchanged in the pH range of 1~12, with the maximum emission wavelength centered around 440–447 nm. At pH > 8.44, the maximum emission wavelength was slightly shifted. The fluorescence intensity at 440 nm exhibited a decrease when acid or base was added to the PT-CQDs solution, indicating that the acid–base environment has an effect on the fluorescence performance of PT-CQDs, with a greater decrease under alkaline conditions. Combined with the results of FT-IR and XPS analysis, we can associate this with the functional groups on the surfaces of PT-CQDs causing disruptions in the surface charges during protonation or deprotonation processes [11,37]. As the pH decreased from 4.82 to 1.58, the amino groups in the PT-CQDs were gradually protonated, and the protons from the protonated nitrogen were introduced into the substantial conjugated nanostructure, causing the fluorescence emission to increase. Due to the proton saturation of the nitrogen sites in PT-CQDs, the fluorescence intensity reached its highest at around pH 4.82 in the original PT-CQDs working solution [9]. In addition, H^+^ can introduce surface defects to PT-CQDs by breaking the passivated OH shell, resulting in fluorescence intensity reductions [38]. Compared to the original PT-CQDs working solution, the fluorescence intensity decreased by approximately 46.2% at pH 11.55. The rapid decrease in fluorescence intensity may be associated with the disruption of hydrogen bonding through deprotonation under alkaline conditions, which can cause irregular energy levels, resulting in the reduction in N-CQDs fluorescence [11,38]. Within the physiological pH range of 7.35~7.45, the fluorescence intensity decreased by 1.3% and remained in the order of 10^5^, indicating that PT-CQDs can be used in the physiological pH range.

The findings of a study of the stability of PT-CQDs at different concentrations of NaCl using fluorescence spectra are shown in Figure 4b. When the concentration of NaCl was 0~2.5 mol·L^−1^, the fluorescence intensity of PT-CQDs at 440 nm slightly decreased by approximately 26% with the increase of NaCl concentration. The fluorescence intensity change F_0_-F (F_0_ represents the intensity of PT-CQDs, and F represents the intensity of PT-CQDs with NaCl) exhibited a linear relationship with the concentration of NaCl. The linear equation was *y* = 222.08194 + 4056.83355*x*, with a correlation coefficient *R*^2^ = 0.99359, indicating that although the fluorescence intensity of PT-CQDs was influenced by the high salt concentration, the effect could be calculated quantitatively.

### 3.3. Selective and Sensitive Fluorescence Sensing Study of PT-CQDs for Fe^3+^ Ions

**Figure 5:**
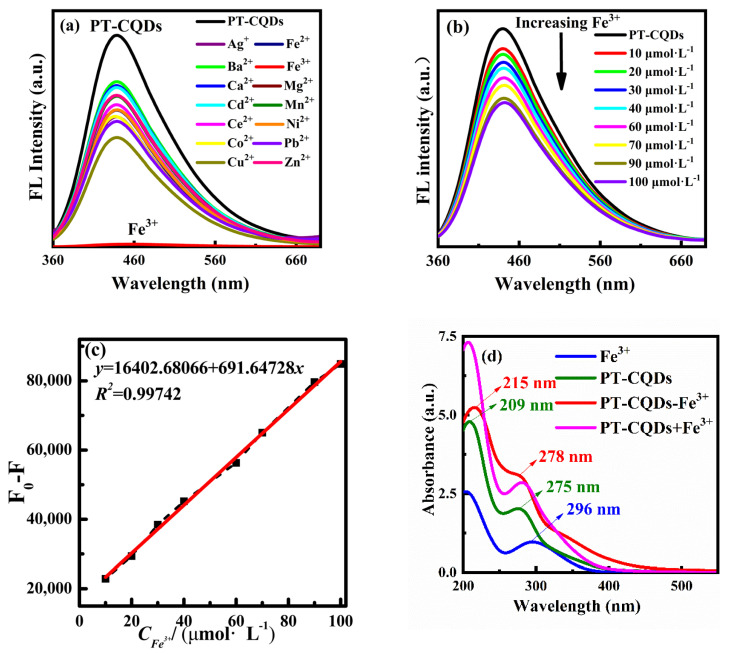
(**a**) Fluorescence spectra of PT-CQDs with different metal ions at the 440 nm emission wavelength. (**b**) Fluorescence spectra of PT-CQDs with different concentrations of Fe^3+^ ions. (**c**) Linear relationship of fluorescence intensity change with Fe^3+^ ion concentration in the range of 10~100 μmol·L^−1^ at 440 nm emission wavelength. (**d**) The UV-visible absorption spectra of PT-CQDs, Fe^3+^, PT-CQDs-Fe^3+^ (3 mL of PT-CQDs and 15 μL of 0.01 mol·L^−1^ Fe^3+^) and PT-CQDs + Fe^3+^ solution.

As PT-CQDs have good fluorescence properties and excellent water solubility, their use as a sensor for metal ions was studied by use of fluorescence spectra. Selectivity is an important indicator for the performance evaluation of fluorescent sensors. In the experiment, fourteen kinds of common metal ions were selected and added to the PT-CQDs working solution, respectively, to investigate the changes in the fluorescence performance of PT-CQDs. As shown in Figure 5a, when the same amounts of different metal ions were added to the PT-CQDs solution, the shape of the fluorescence spectra did not change significantly, and the intensity changed slightly, except when adding Fe^3+^ ions. It can be seen that Fe^3+^ ions are unique, as they almost completely quenched the fluorescence of PT-CQDs. It is thus indicated that PT-CQDs have good selectivity for Fe^3+^ ions. Photos of the PT-CQDs with different metal ions under ambient and UV light are shown in Figure S1 (Supporting Information). The decrease in fluorescence intensity may be caused by the functional groups on the surface of PT-CQDs, such as amino, carboxyl and hydroxyl groups, which are easily chelated with metal ions to undergo electron transfer.

To further demonstrate the influence of interfering ions on the sensing ability of a PT-CQDs-based fluorescent probe for Fe^3+^ ions, the results of the study are shown in Figure S2 (Supporting Information). The results reveal that when the same amounts of Fe^3+^ ions were added to the mixed solution of PT-CQDs and interfering ions, the fluorescence intensities were all almost quenched. These phenomena are consistent with those seen for PT-CQDs in the presence of Fe^3+^ ions only. Thus, the interfering metal ions have no significant effect on the detection of Fe^3+^ ions by PT-CQDs.

The sensitive detection of Fe^3+^ ions by PT-CQDs is shown in Figure 5b. It can be seen that the fluorescence intensity of PT-CQDs gradually decreases with the increasing concentration of Fe^3+^ ions in the range of 0~100 μmol·L^−1^. Figure 5c shows that the change in fluorescence intensity F_0_-F versus Fe^3+^ concentration in the range of 10~100 μmol·L^−1^ at the emission wavelength of 440 nm exhibited a good linear relationship (F_0_ represents the intensity of PT-CQDs, and F represents the intensity of PT-CQDs with Fe^3+^). The linear equation used was *y* = 691.64728 *x* + 16,402.68066, with a correlation coefficient *R^2^* = 0.99742. A new method for the determination of Fe^3+^ ions’ concentration is established based on this study. The LOD for Fe^3+^ ions was calculated to be 2.7 μmol·L^−1^, which is lower than the maximum permissible limit of Fe^3+^ ions in drinking water set by the World Health Organization (5.36 μmol·L^−1^) [18,23], indicating that the synthesized PT-CQDs have high detection sensitivity for Fe^3+^ ions.

### 3.4. Sensing Mechanism of PT-CQDs for Fe^3+^ Ions

We preliminarily explored the mechanism of action PT-CQDs used as a sensor for Fe^3+^ ions in an original working solution without pH adjustment. In general, fluorescence quenching follows five major mechanisms, namely, inner filter effect (IFE), dynamic quenching, static quenching, Forster energy resonance transfer (FRET) and photo-induced electron transfer (PET) [39,40]. Figure 5d shows there was obvious absorption of Fe^3+^ in the range of 200–400 nm. Figure 3b shows that Fe^3+^ ions showed strong absorption in the range of 200–350 nm, while it partially overlaps with the excitation spectrum of PT-CQDs in the range of 300–430 nm and emission wavelengths in the range of 360–400 nm. We thus infer that the inner filter effect (IFE) occurred when Fe^3+^ ions coexisted with PT-CQDs [41,42]. Figure 5d shows that after the addition of Fe^3+^ ions, the two UV–visible absorption peaks of PT-CQDs showed slight red shifts, and the absorbance increased. The absorption spectra of the PT-CQDs-Fe^3+^ system were significantly different from the algebraic and additive absorption spectra of PT-CQDs and Fe^3+^ ions (PT-CQDs + Fe^3+^). This indicates that there was an interaction between PT-CQDs and Fe^3+^ ions. To further study the sensing mechanism, the fluorescence decay spectra of PT-CQDs in the absence and presence of Fe^3+^ were assessed. For static quenching, average fluorescence lifetime values remained unchanged, and the formation of the ground–state complex was shown to result in a change in the absorption spectrum of the CQDs. As shown in Figure S3, the average lifetime values of PT-CQDs and PT-CQDs-Fe^3+^ were 1.67 ns, 1.63 ns (60 μmol·L^−1^ Fe^3+^) and 1.49 ns (0.33 mmol·L^−1^ Fe^3+^), respectively. The small change in fluorescence lifetimes after the addition of Fe^3+^ indicates that the mechanism was not FRET, PET or dynamic quenching, since all of these cause significant changes in average lifetime [39,43]. We have thus inferred that the fluorescence quenching of PT-CQDs by Fe^3+^ ions may be due to IFE and static quenching.

From the analysis of XPS and FT-IR spectra, we can see that a large number of hydroxyl, amino and carboxyl groups on the surfaces of PT-CQDs may bind to the d-orbital of Fe^3+^ via electron transfer and chelate to inhibit the fluorescence of PT-CQDs [10]. As such, zeta potential characterization was performed to determine the surface electronic charge of the PT-CQDs. Zeta potential is a measure of the electrostatic potential at the particle’s surface, and indicates the stability of the solution system. Table S2 shows the zeta potentials of PT-CQDs with and without Fe^3+^ at different pH values. The zeta potentials of PT-CQDs in the range of pH 2.53~11.55 were all negative, indicating that the surfaces of PT-CQDs had negative charge. After the addition of 60 μmol·L^−1^ Fe^3+^, the zeta potentials within the pH range of 4.82~11.55 were all negative, indicating that the system of PT-CQDs-Fe^3+^ also has negative charges. This may be because of the dense electron cloud concentrating on the PT-CQDs in the presence of functional groups, like hydroxyl and carbonyl groups, on the surface [23]. When the –OH or –COOH were coordinated with Fe^3+^ ions to form a ground–state complex, the oxygen obtained a positive charge with increasing zeta potential [44,45]. When the pH was below 2.53 and above 10.49, the zeta potentials of PT-CQDs-Fe^3+^ were higher than that of PT-CQDs. This may have been caused by the chelation of Fe^3+^ and carboxyl groups. With a pH from 4.82~7.55, the zeta potentials of PT-CQDs-Fe^3+^ were lower than those of PT-CQDs. We conclude that chelation between –OH or –COOH and Fe^3+^ did not affect the fluorescence intensity of PT-CQDs-Fe^3+^ in the pH range of 4.82~7.55.

Figure S4 shows the FT-IR spectra of PT-CQDs with Fe^3+^ ions after drying with an infrared lamp. Compared with the PT-CQDs, the FT-IR spectral characteristics of PT-CQDs were changed with the addition of Fe^3+^ ions. The peak at approximately 612 cm^−1^ was converted into a shoulder peak after the addition of Fe^3+^ ions. The intensities of the peaks at 1646 cm^−1^ (C=C, C=N and C=O), 1616 cm^−1^ (O-C=O) and 1044 cm^−1^ (C-N) in the FT-IR spectrum were all increased after the addition of Fe^3+^ ions. This indicates that there was an interaction between PT-CQDs and Fe^3+^ ions. As reported [46,47], when Fe^3+^ ions bind to the −COOH and −OH groups of PT-CQDs, the absorbance levels of C–H, C=O, C=C, and C–O bonds are weakened. Thus, the authors also conclude that chelation between –OH or –COOH and Fe^3+^ did not occur in the original working solution (pH = 4.82). This result is consistent with those from zeta potential studies.

In summary, the fluorescence quenching mechanism of PT-CQDs by Fe^3+^ ions may operate via IFE and static quenching in the original working solution (pH = 4.82). The Fe^3+^ ion sensing properties and mechanisms of the other nanoprobes used as fluorecence sensors are summarized in Table S1. The results denote that the developed method exhibits the good selective and sensitive detection of Fe^3+^ ions compared with the other reported methods.

### 3.5. Application of Anti-Blue Light PT-CQDs/PVA Flims

Another important application of the PT-CQDs/PVA film is as an efficient UV and HEBL shield. As mentioned in Figure 3b, the UV-Vis absorption spectrum of the PT-CQDs solution showed strong absorption at 209 nm, a shoulder peak at 275 nm, and a tailing peak (~450 nm), indicating that the PT-CQDs can absorb UV and HEBL simultaneously. The PT-CQDs can absorb HEBL due to the tailing peak, which possibly arises from their defect states. Different types of nitrogen- and oxygen-related functional groups in PT-CQDs and π/n−π* transitions in carbon cores can be identified from XPS and FTIR analyses, together with the UV−Vis analysis. These introduce some defects, which contribute to the broad absorption of HEBL. Therefore, this absorption by PT-CQDs is due to the presence of surface-functionalized groups and the level of defects in the PT-CQDs, which have a robust ability to absorb UV and HEBL.

**Figure 6:**
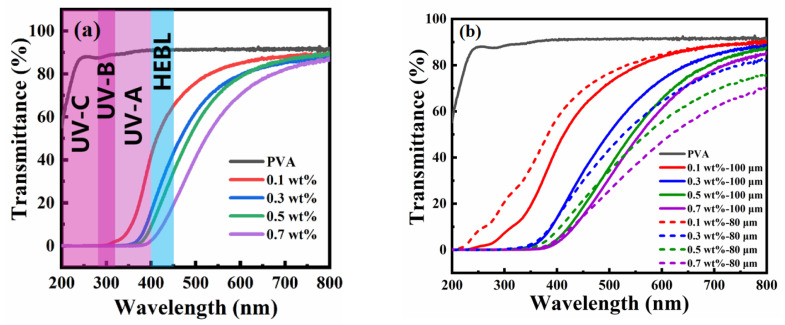

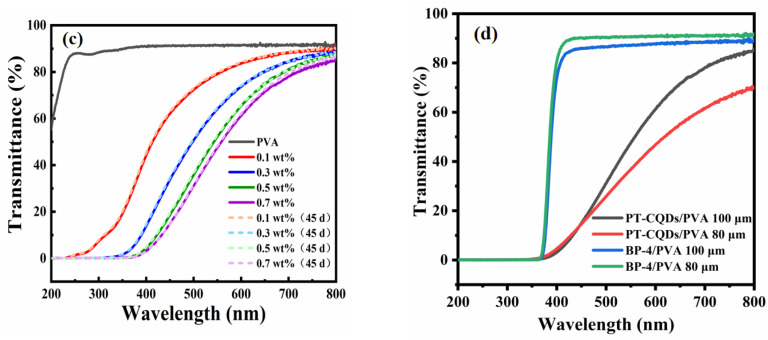
UV-Vis transmittance spectra of PT-CQDs/PVA films with different concentrations of PT-CQDs and pure PVA films (**a**); PT-CQDs/PVA films with different thickness (80 μm, 100 μm) (**b**); PT-CQDs/PVA films with the thickness of 100 μm and different concentrations at the beginning and after 45 days (**c**); PT-CQDs/PVA films and BP-4/PVA films with the proportion of 0.7 wt. % and different thickness of 80 μm and 100 μm (**d**).

To evaluate the films’ effectiveness in blocking UV and HEBL, we prepared PT-CQDs/PVA films and analyzed the UV-Vis transmittance spectra of PT-CQDs/PVA films with different concentrations of PT-CQDs (0.1, 0.3, 0.5, 0.7 wt. %), different thicknesses (80 μm, 100 μm), and bare PVA film, as depicted. As shown in Figure 6a, when the thickness of the PT-CQDs/PVA film and bare PVA film was 100 μm, PVA film was highly transparent in the UV-A and -B and visible regions (90%), indicating that bare PVA film is not suitable for blocking UV-A or -B or HEBL. By incorporating different wt. % of PT-CQDs into the PVA films, it was found that they were able to absorb all UV-A and -B and HEBL. As the PT-CQD loading increased from 0.1 wt. % to 0.7 wt. %, the UV-A/-B transmission approached zero, while the visible light transparency remained higher. The 0.1 wt. % PT-CQD film blocked ∼45% of UV-A, ∼0% of UV-B, ∼67% of HEBL and 80% of visible light (540 nm). However, the 0.7 wt. % PT-CQD film perfectly blocked UV-A and UV-B, and 20% of HEBL, along with showing ∼60% transparency for visible light. These films are sufficiently transparent to visible light and block most of the harmful UV and HEBL. In order to further explore the effects of different thicknesses on the films’ performances, PT-CQDs/PVA films with the thickness of 80 μm were also prepared, and their transmittance spectra are investigated in Figure 6b. Under a low concentration of PT-CQDs (0.1 wt. %), compared with the PT-CQDs/PVA film (80 μm), the PT-CQDs film (100 μm) better absorbed UV-A and -B. When the proportion of PT-CQD was gradually increased from 0.3 wt. % to 0.7 wt. %, the PT-CQD film (80 μm) and PT-CQDs film (100 μm) showed a similar capacity for blocking UV and HEBL; however, under visible light (>540 nm), the PT-CQDs film (100 μm) showed higher transparency. In a word, the PT-CQDs film (100 μm) showed a better performance than the PT-CQDs film (80 μm). Figure S5 shows photographs of PT-CQDs/PVA films containing various wt. % (0.1, 0.3, 0.5, 0.7) and with different thicknesses (80 μm, 100 μm). PT-CQDs/PVA films were transparent, and the color of the PT-CQDs/PVA become darker as the concentration of doped PT-CQDs increased, but this did not affect the transparency of the films. If we explore PT-CQDs films with higher concentrations and thicknesses, these properties may disrupt transparency due to the original color of the PT-CQDs, which is dark brown. To evaluate the stability of PT-CQDs/PVA films in real-world applications, long-term stability tests of films were conducted and the results are displayed in Figure 6c. Remarkably, the transmittance spectra of PT-CQDs films remained unchanged for 45 days, implying the long-term anti-blue light properties of PT-CQDs films. Furthermore, the blocking capacity of PT-CQDs films was compared with that of commercially available anti-blue light lenses (2-hydroxy-4-methoxy benzophenone-5-sulfonic acid, BP-4), and the results are shown in Figure 6d. With the same concentration of 0.7 wt. %, both the BP-4-doped PVA films and PT-CQDs/PVA film could absorb UV-A, -B and -C regions, but in the range >450 nm, the PT-CQDs/PVA film could only absorb HEBL, contributing to its wider application.

**Figure 7:**
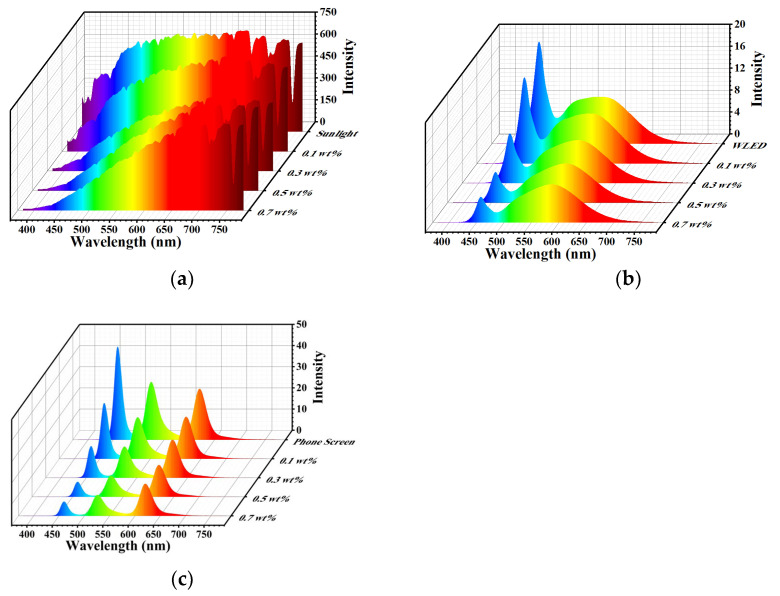
Three-dimensional spectrograms of PT-CQDs/PVA films with different concentrations derived from sunlight (**a**), WLED (**b**) and mobile phone (**c**) sources.

The abilities of the films to block harmful blue light were investigated. The most common light sources, such as sunlight, mobile phones and WLEDs, were selected because they emit UV and HEBL lights. The three-dimensional spectrograms of PT-CQDs/PVA films with different concentrations from different sources are shown in Figure 7. The spectral characteristics of the CIE chromaticity coordinates, color rendering index (CRI), correlation color temperature (CCT), and RGB ratio of the films blocking sunlight, WLED and mobile phone signals are summarized in Table 1, Tables S3 and S4. As shown in Figure 7a, when the films’ thickness was kept at 100 μm, as the concentration of the films increased from 0.1 wt. % to 0.7 wt. %, the intensity of the absorbed light (<450 nm) was significantly attenuated, but the intensities of blue, yellow and red light in the 500–700 nm band were slightly changed. More detailed changes can be observed in Table 1. For instance, the proportion of blue light was reduced from 6.1% to 2.8% and the proportion of green light was slightly reduced from 75.4% to 71.9%, but the proportion of red light was increased from 18.5% to 25.3%. Meanwhile, the CRI and CCT parameters were gradually simultaneously reduced. This suggests that PT-CQDs/PVA films could absorb the harmful blue light emitted from sunlight and convert it into harmless red light. The same phenomenon can also be observed in relation to other light sources (WLEDs, mobile phones). Although all PT-CQDs/PVA films showed a good ability to block the harmful blue light emitted from the light sources of sunlight, WLEDs and mobile phones, there are slight differences in their practical applications. For example, when absorbing more harmful blue light from displays such as mobile phones and WLEDs, the CIE chromaticity coordinates are changed and the quality of the color image is reduced, but this does not have a significant effect on the light sources. Therefore, it is essential to satisfy the needs of different applications by regulating the concentrations of the films.

## 4. Conclusions

In conclusion, new carbon quantum dots (PT-CQDs), with the leaves of Peperomia tetraphylla as a carbon source, were synthesized and characterized by a simple one-step hydrothermal method. This method not only effectively utilizes biological waste, but also conforms to the concept of sustainable development. The leaves of Peperomia tetraphylla are a rich carbon source. The whole process was green and environmentally friendly, the cost was very low, and the synthesis method was simple and easy to complete. The obtained PT-CQDs exhibited strong fluorescence emission at 450 nm, an average diameter of about 4.73 ± 1.02 nm, good water solubility, and high stability in the physiological pH range. XPS and FT-IR analyses showed that the surfaces of PT-CQDs contained abundant functional groups, such as amino, hydroxyl and carboxyl groups. PT-CQDs have been applied as sensors for Fe^3+^ ions. Their fluorescence performance assessment showed a good linear relationship between fluorescence intensity changes and the concentration of Fe^3+^ ions in the range of 10~100 μmol·L^−1^, and the LOD was 2.7 μmol·L^−1^. PT-CQDs were also here embedded into PVA matrix films to produce novel PT-CQDs/PVA films with excellent UV and HEBL shielding capacities. They achieved high blocking rates of 100% for UV-A and UV-B and 80% for HEBL, while maintaining adequate transmission in the visible range of 500–750 nm. The concentrations of the composites could be controlled to achieve the desired blue light blocking rate. Additionally, the composites were able to absorb blue light and convert it into other forms of light. These properties suggest that PT-CQDs have potential applications in anti-blue light technologies and fluorescence sensors.

## Figures and Tables

**Table 1:** The spectral characteristics of different concentrations of PT-CQDs/PVA films under sunlight.

Sunlight		CIE Coordinates	Ra	CCT (K)	Red (%)	Green (%)	Blue (%)
	blank	(0.3529, 0.3618)	98.8	4756	18.5	75.4	6.1
PT-CQDs	0.1 wt. %	(0.3805, 0.3854)	97.9	4059	20.0	74.9	5.2
	0.3 wt. %	(0.4151, 0.4107)	96.2	3452	21.9	74.0	4.1
	0.5 wt. %	(0.4475, 0.4260)	95.1	3000	24.2	72.7	3.2
	0.7 wt. %	(0.4606, 0.4291)	95.0	2831	25.3	71.9	2.8

## Data Availability

Data are contained within the article and Supplementary Materials.

## Supplementary Materials

The following supporting information can be downloaded at: https://www.mdpi.com/article/10.3390/nano15060436/s1, Figure S1: The photographs of 2 mL PT-CQDs working solution with 1 mL of 10 mmol L^−1^ different metal ions (0 represents PT-CQDs, 1 to 14 represent PT-CQDs mixed with metal ions in turn: Ag^+^, Ba^2+^, Ca^2+^, Cd^2+^, Ce^2+^, Co^2+^, Cu^2+^, Fe^2+^, Fe^3+^, Mg^2+^, Mn^2+^, Ni^2+^, Pb^2+^, Zn^2+^) under ambient and UV light; Figure S2: (a) Fluorescence emission spectra and (b) intensity changes in the presence of interfering metal ions with the addition of Fe^3+^ at 440 nm (F represent the fluorescence intensities in the presence of Fe^3+^ in the system of PT-CQDs-metal ion, F_0_ represent the fluorescence intensity of PT-CQDs. Inset photographs displays the color variation of PT-CQDs-Fe^3+^ with and without interfering metal ions under ambient and UV light, 0 represents PT-CQDs, 9 represents PT-CQDs-Fe^3+^, 1 to 14 represent PT-CQDs-Fe^3+^ sequentially mixed with interfering ions: Ag^+^, Ba^2+^, Ca^2+^, Cd^2+^, Ce^2+^, Co^2+^, Cu^2+^, Fe^2+^, Fe^3+^, Mg^2+^, Mn^2+^, Ni^2+^, Pb^2+^, Zn^2+^); Figure S3: Fluorescence decay spectra of the PT-CQDs with and without Fe^3+^; Figure S4: FT-IR spectra of PT-CQDs with Fe^3+^ ions after drying with an infrared lamp (2 mL PT-CQDs working solution mixed with 1 mL 10 mmol·L-1 Fe^3+^); Figure S5: Photographs of PT-CQDs/PVA films containing various wt% (0.1, 0.3, 0.5, 0.7) and different thickness (80 μm, 100 μm); Table S1: Comparison of different fluorescent nanoprobe sensors for detection of Fe^3+^ ions. Table S2: The average zeta potentials of PT-CQDs with and without Fe^3+^ (60 μmol L^−1^) at different pH values; Table S3: The spectral characteristics of different concentrations of PT-CQDs/PVA films covering WLEDs; Table S4: The spectral characteristics of different concentrations of PT-CQDs/PVA films covering mobile phones.
