# Supplementary material for: Dual Roles of Carbon Quantum Dots from Green Carbon Sources: A Fluorescence Sensor for Fe3+ Ions, UV and High-Energy Blue Light Screening
Source: Nanomaterials (Basel). 2025 Mar 12;15(6):436. doi: 10.3390/nano15060436 (PMC11945768; doi:10.3390/nano15060436)
Supplement: Supplementary file 1 [file nanomaterials-15-00436-s001.zip › nanomaterials-3474988-supplementary.pdf]

Supporting Information of

Dual Roles of Carbon Quantum Dots from Green Carbon Sources: A Fluorescence Sensor for Fe^{3+} Ions, UV and High-Energy Blue Light Screening

Lina Zhong, Chang Sun, Xiaomin Zhao and Qinghua Zhao *

College of Materials Science and Engineering, Huaqiao University, Xiamen 361021, China; lnzhong@hqu.edu.cn (L.Z.); 22013081053@stu.hqu.edu.cn (C.S.); 15356@hqu.edu.cn (X.Z.)

* Correspondence: qhzhao@hqu.edu.cn

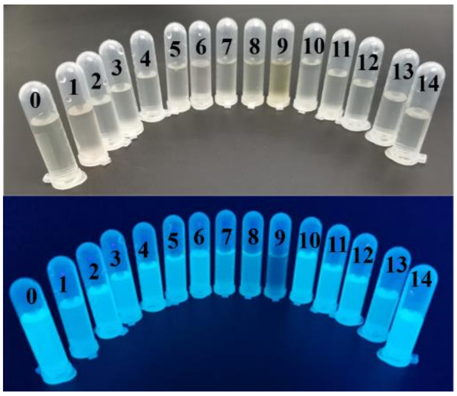

Figure S1. The photographs of 2 mL PT-CQDs working solution with 1 mL of 10 mmol·L^{-1} different metal ions (0 represents PT-CQDs, 1 to 14 represent PT-CQDs mixed with metal ions in turn: Ag^{+}, Ba^{2+}, Ca^{2+}, Cd^{2+}, Ce^{2+}, Co^{2+}, Cu^{2+}, Fe^{2+}, Fe^{3+}, Mg^{2+}, Mn^{2+}, Ni^{2+}, Pb^{2+}, Zn^{2+}) under ambient and UV light.

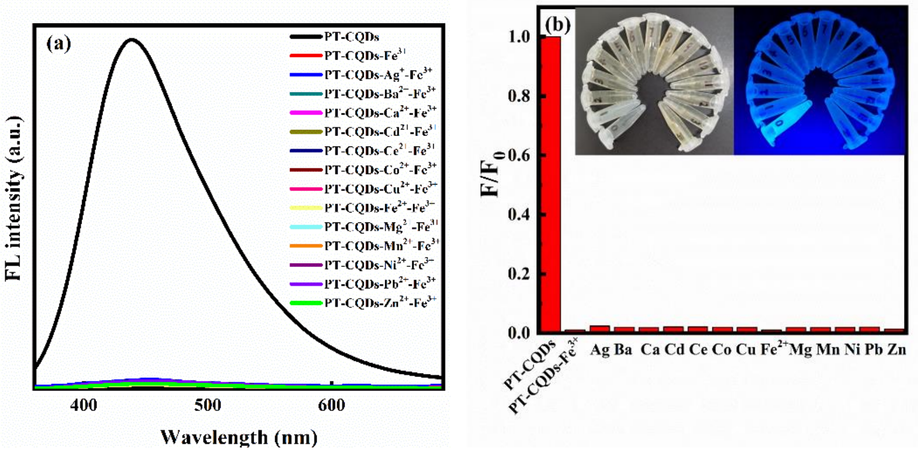

Figure S2. (a) Fluorescence emission spectra and (b) intensity changes in the presence of interfering metal ions with the addition of Fe^{3+} at 440 nm (F represent the fluorescence intensities in the presence of Fe^{3+} in the system of PT-CQDs-metal ion, F_0 represent the fluorescence intensity of PT-CQDs. Inset photographs displays the color variation of PT-CQDs-Fe^{3+} with and without interfering metal ions under ambient and UV light, 0 represents PT-CQDs, 9 represents PT-CQDs-Fe^{3+}, 1 to 14 represent PT-CQDs-Fe^{3+} sequentially mixed with interfering ions: Ag^{+}, Ba^{2+}, Ca^{2+}, Cd^{2+}, Ce^{2+}, Co^{2+}, Cu^{2+}, Fe^{2+}, Fe^{3+}, Mg^{2+}, Mn^{2+}, Ni^{2+}, Pb^{2+}, Zn^{2+}).

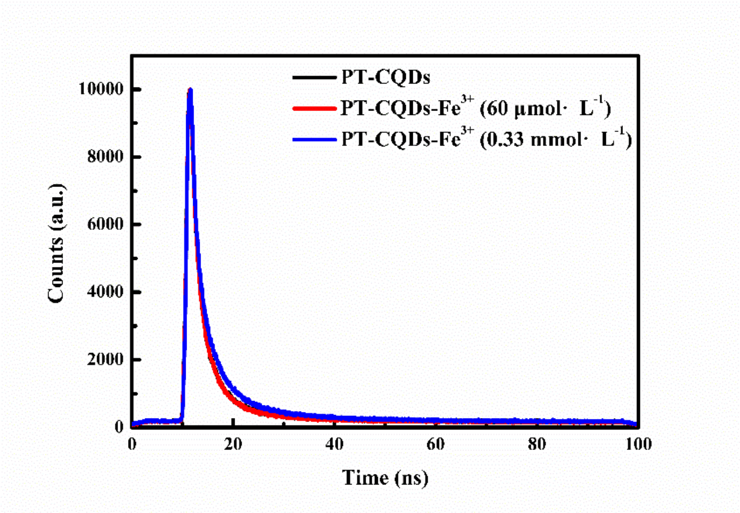

Figure S3. Fluorescence decay spectra of the PT-CQDs with and without Fe^{3+}.

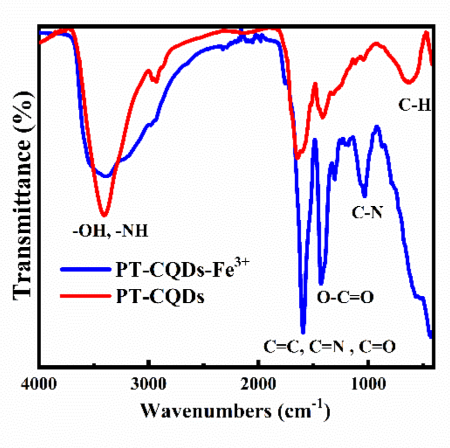

Figure S4. FT-IR spectra of PT-CQDs with Fe^{3+} ions after drying with an infrared lamp (2 mL PT-CQDs working solution mixed with 1 mL 10 mmol·L^{-1} Fe^{3+}).

Table S1. Comparison of different fluorescent nanoprobe sensors for detection of Fe^{3+} ions.

Raw materials	Sensing mechanism	Liner range (μmol·L^{-1})	LOD (μmol·L^{-1})	Ref.
Morinda coreia fruits	Static quenching and dynamic quenching	0-250	1.32	[11]
Cauliflower and Garlic	IFE, static quenching	10-400	3.29	[13]
Lentinus polychrous Lèv. mushrooms	IFE,static quenching and dynamic quenching	0-2000	16	[42]
Citric acid, urea and bulk g-C_3N_4	Dynamic quenching	30-100	3.7	[9]
Citric acid and ethylenediamine	Dynamic quenching	0-2	0.07	[24]
Citric acid and glutamine	Static quenching	2-40	1.05	[40]
Gluthamic acid and ethylenediamine	Static quenching	8-80	3.8	[37]
Leaves of Peperomia tetraphylla	IFE and static quenching	10-100	2.7	This work

Table S2. The average zeta potentials of PT-CQDs with and without Fe^{3+} (60 μmol·L^{-1}) at different pH values.

pH	Average zeta potential of PT-CQDs (mV)	Average zeta potential of PT-CQDs-Fe^{3+} (mV)
1.58	1.30	10.82
2.53	-0.61	7.72
4.82	-5.23	-10.20
6.49	-14.47	-15.37
7.55	-16.97	-18.23
10.49	-18.97	-18.73
11.55	-21.03	-20.70

Table S3. The spectral characteristics of different concentrations of PT-CQDs/PVA films covering WLEDs.

WLEDs		CIE coordinates	Ra	CCT (K)	Red (%)	Green (%)	Blue (%)
	blank	(0.3072，0.3262)	81.9	6839	13.1	80.6	6.4
	0.1 wt%	(03258，0.3491)	81.3	5788	14.1	80.4	5.4
PT-CQDs	0.3 wt%	(0.3636，0.3874)	77.0	4543	15.6	80.3	4.2
	0.5 wt%	(0.3950，0.4145)	75.1	3900	17.0	79.8	3.2
	0.7 wt%	(0.4072，0.4235)	74.2	3702	17.6	79.5	2.9

Table S4. The spectral characteristics of different concentrations of PT-CQDs/PVA films covering mobile phones.

Mobile phones screen		CIE coordinates	Ra	CCT (K)	Red (%)	Green (%)	Blue (%)
	blank	(0.2922，0.3054)	65.0	8218	23.7	70.2	6.1
	0.1 wt%	(0.3193，03299)	60.9	6150	26.1	69.0	5.0
PT-CQDs	0.3 wt%	(0.3614，0.3627)	57.7	4475	29.7	66.7	3.7
	0.5 wt%	(0.4116，0.3935)	55.7	3388	33.8	63.7	2.4
	0.7 wt%	(0.4164，0.3957)	55.6	3309	34.2	63.4	2.4

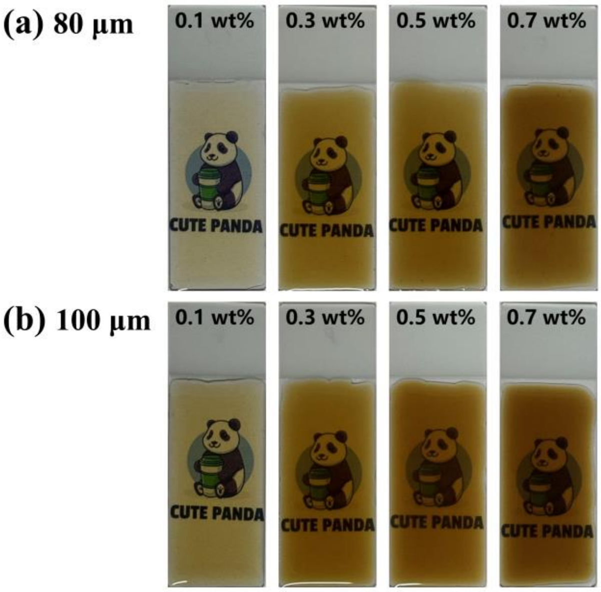

Figure S5. Photographs of PT-CQDs/PVA films containing various wt% (0.1, 0.3, 0.5, 0.7) and different thickness (80 μm, 100 μm).
